# Long-term outcomes and propensity score matching analysis: rectal cancer resection for patients with elevated preoperative risk

**DOI:** 10.18632/oncotarget.13827

**Published:** 2016-12-09

**Authors:** Hao Feng, Tobias S. Schiergens, Zhi-hai Mao, Jingkun Zhao, Xiaohui Shen, Ai-Guo Lu, Wolfgang E. Thasler

**Affiliations:** ^1^ Department of Digestive Surgery, Ruijin Hospital, Shanghai Jiao-tong University School of Medicine, Shanghai, China; ^2^ Department of General, Visceral, Transplant, and Vascular Surgery, University Hospital of Ludwig Maximilian University of Munich, Munich, Germany; ^3^ Department of General and Visceral Surgery, Red Cross Hospital of Munich, Munich, Germany

**Keywords:** laparoscopy, rectal cancer, operative risk, Cr-POSSUM system, complications

## Abstract

**BACKGROUND:**

It is still controversial about the treatment strategy for rectal cancer patients with elevated operative risk and elder rectal cancer patients.

**METHODS:**

This study presented a retrospective single center experience in rectal cancer proctectomy for high operative risk patients. High operative risk patient was defined as Cr-POSSUM > 5% combined with associated risk factors. 220 in 1477 consecutive patients met the inclusion criteria.

**RESULTS:**

132 patients were selected (66:66) after propensity score matching. The total complication rate between conventional open rectal resection (71 %) and laparoscopic surgery (41%) was significantly different (*p* = 0.0005). There is a significantly positive correlation between open surgery and advanced Dindo Classification (*p* = 0.02). Cr-POSSUM is positively correlated with Dindo Classification (*p* = 0.01). There was no significant difference in survival rate among stage I∼II, different age groups or different Cr-POSSUM score sub-groups. However, stage III-IV tumor patients in laparoscopic group experienced improved overall survival rate. (*p* < 0.0001). For patients with preoperative pulmonary or renal disease, patients in laparoscopic group also had better long term prognosis (*p* = 0.03, *p* = 0.049).

**CONCLUSIONS:**

The results demonstrate the potential advantages of laparoscopic rectal cancer resection for high operative risk patients, especially for the patients with preoperative respiratory or renal disease and stage III cancer.

## INTRODUCTION

Rectal cancer is associated with substantial morbidity and mortality, especially in elder patients and those with co-morbidities. Outcome after these surgeries depends both on modifiable factors, such as perioperative medical care, and on physiological tolerance of surgical trauma. Over the last two decades, we have seen a continuous improvement of the quality of laparoscopic surgery in rectal cancer, especially in specialized centers with longstanding experience and high annual volumes. Several studies that compared laparoscopic and conventional open resection for rectal cancer show no difference with respect to local recurrence or overall and disease-free survival after 3, 5 [1, 2] even 10 years [3], respectively. More recently, long-term data including the MRC CR07 [4], MRC CLASICC trial, Comparison of Open versus laparoscopic surgery for mid or low Rectal cancer After Neoadjuvant chemoradiotherapy (COREAN) trial [5], the Colorectal cancer laparoscopic or Open Resection (COLOR II) trial [6] have released long-term survival rates. Though some of the randomized control trials have included patients with elevated preoperative risk (American Society of Anesthesiologists classification 3 and 4), these patients were generally recruited to clinical trials less often than younger patients and therefore are under-represented in publications about cancer treatment [7]. Because of this heterogeneous, can these recommendations from major studies, such as laparoscopic rectal operations are safe and sound, be extrapolated to the fragile subset of patients with more comorbidity or do they need to be modified? The aim of this study is to analysis the survival and outcomes in patients with rectal cancer associated with high operative risk in conventional open rectal resection group (OpS) and laparoscopic rectal resection group (LaPS).

## MATERIALS AND METHODS

### Patients

This study included all 1477 consecutive patients undergoing radical surgical resection for rectal cancer in a tertiary referral teaching hospital - Shanghai Ruijin Hospital between September 2007 and Nov 2011. 220 patients were considered with high operative risk. Patients were admitted to Gastrointestinal Surgical Centre or Minimally Invasive Surgical Centre. Both centers belong to Department of General Surgery. The operative conditions, anesthesia management as well as perioperative management were at the same level. Both surgical teams had the same operative quality of rectal cancer. Emergency protectomy was excluded.

### Diagnoses and tumor stage

The diagnoses were made preoperatively and then confirmed by postoperative pathology. The tumor node metastasis (TNM) staging of colon and rectal cancer system (American Joint Committee on Cancer Manual, 7th edition) was used. The criteria for neoadjuvant radiochemotherapy were patients with rectal cancer of the lower and middle third of the rectum and suspected T3 or T4 tumors and patients with pathological lymph nodes as demonstrated by CT or MRI-scan.

### Surgical procedures and quality control

Patient demographics were extracted routinely by trained registrars from the hospital records. Patients were assigned preoperatively to the laparoscopic or open approach based on clinical criteria and imaging, including chest radiograph, abdominal computed tomography, and colonoscopy etc. Patients’ preference had also been considered. Conversion cases were deemed necessary remained in the laparoscopic surgery group for all outcomes by intention-to-treat analysis. The preoperative preparation and the techniques of the procedures were described previously. With our experience from open total mesorectal excision, laparoscopic surgery was performed according to the same oncologic principles [8, 9]. Briefly, laparoscopic surgery was done with five trocars, the rectum was mobilized with monopolar cautery or an ultrasonic scalpel, dissecting between the visceral and parietal pelvic fascia without injuring the hypogastric nerves. Laparoscopic and open procedures were performed by four senior surgeons with their specialist team from the division of Gastrointestinal Surgery or division of Minimally Invasive Surgery in Ruijin Hospital. In the LapS group, surgery was performed by a systemic team of surgeons with abundant experience and expertise in conventional colorectal surgery and laparoscopic skills. In the OpS group, another fixed group of experienced surgeons specializing in colorectal surgery executed the surgery. [9]

### Statistical methods

Analyses were performed with Stat View 5.0 for Windows (SAS Institute Inc., Cary, NC, USA). The Х2 test or Fisher's exact test was applied to analyze the categorical variables. The results were subjected to a nonparametric Mann-Whitney U test. A Student's t-test was also used to analyze the intragroup differences. The Kaplan-Meier method was used to analyze the overall survival of patients; the log-rank test was used to compare patient survival between groups. Cox-regression model was used for multivariate analysis. Logistic regression was used to analyze the correlation of Cr-POSSUM and Dindo-Demartines-Clavien Classification. P < 0.05 was regarded as statistically significant.

### Propensity score matching

Propensity score matching was applied to reduce the effect of treatment selection bias and potential confounding effect, thereby creating a quasi-randomized experiment. This matching is done using a generalized SAS macro that matches Ops to LapS at a 1:1 ratio, using an algorithm to maximize the number of propensity score match. Patients were selected based on this score calculating for baseline characteristics; that is age, gender, tumor size, tumor location, tumor stage, Cr-possum value and radiochemotherapy at baseline in patients.

### Risk evaluation

Patients with a predicted Colorectal Physiologic and Operative Severity Score for the enumeration of Mortality and Morbidity (Cr-POSSUM) ≥5% OR criteria below [10] were managed as ‘high operative risk’:

1. Aged > 60 years
  PLUS undergoing re-do surgery
  OR have acute or chronic renal impairment (sCr > 130 μmol/L)
  OR have diabetes mellitus
  OR are strongly suspected clinically to have any significant risk factor for the cardiac or respiratory disease. (e. g. chronic obstructive pulmonary disease, history of ischemic heart disease, congestive heart failure, arrhythmias, angina pectoris, or cardiac risk index > 12 etc.)
2. have shock of any cause, any age group.
  Cr-POSSUM scores were calculated for each patient retrospectively from their medical records. The calculating software is freely available on the internet (http://www.riskprediction.org.uk/index-cr.php, Risk Prediction in Surgery)

## RESULTS

There was no significant difference between each group concerning the age (69±11.2 vs 68±12.1 years old, p = 0.5907). The Body mass index (BMI) were 28.1kg/m2 and 27.9kg/m2 (p = 0.437). The tumor size was 3.60±1.58cm and 3.57±0.84cm, respectively (p = 0.916), and located in 6.18cm and 6.36cm from the anal verge. The tumor stage, postoperative radiochemotherapy, circumferential resection margin ( < 2mm) positivity (LapS 1of 66 [2%] vs OpS 1 of 66 [2%]), distal margin, macroscopic completeness of the resection (incomplete rate: LapS 9% vs OpS 10%), locoregional recurrence rate (LapS 4of 66 [6%] vs OpS 5 of 66 [8%]) did not differ between laparoscopic and open surgery groups.

### Operative risk

The distribution of ages and Cr-POSSUM were showed in Table 1. 37 patients (56%) in OpS group were with a Cr-POSSUM score ≥10%, 13 patients (20% in total) of which were with a Cr-POSSUM score ≥20%; while in LapS group, the amount of patients with score above 10% and 20% were 30 patients (45%) and 15 patients (22%), respectively. Concerning the 4 patients whose scores were below 5% in OpS group, three patients were older than 50 years old with pulmonary dysfunction, one patient was 59 years old undergoing re-do surgery. In LapS group, three in five patients were beyond 50 years old combining with pulmonary dysfunction; one was with chronic renal impairment; one patient experienced re-do surgery. In total, there were 3 and 2 patients in each group underwent re-do surgery, eighteen and twelve patients suffered from acute or chronic renal impairment, thirteen and eight patients were suffering from diabetes mellitus in OpS and LapS group, respectively. 50%, 50% patients in OpS group and 32%, 33% patients were suffering from Cardiac and respiratory disease, respectively. 3% patients in the open surgery group have cerebrovascular disease. Generally speaking, there was no significant difference between the two groups in preoperative risk.

**Table 1:** The patient demographics and histopathological tumor assessment

Clinical or pathologic feature	OpS (*n*= 66)	LapS (*n*= 66)	*P*-value	95%CI	
**Sex ratio ( Male: Female)**	45:22	46:21	0.85	-0.1794-0.1491	
**Age (years)**	69±11.2	68±12.1	0.59	-2.984-5.196	
**Body mass index (kg/m2)**	28.1	27.9	0.44		
**pTumor stage (AJCC)**					
I	18	17			
IIA	17	10			
IIIB	6	12			
IIIA	3	5	0.85	-0.2982-0.3588	
IIIB	10	12			
IIIC	4	7			
IV	8	3			
**Lymph node metastasis**					
N0	42	40	0.81		
N1	15	17			
N≥2	9	9			
**Tumor size (diameter, cm)**	3.60±1.58	3.57±0.84	0.92	-0.3812-0.4239	
**Tumor location from anal verge (cm)**	6.18±1.94	6.36±2.06	0.54	-0.5259-0.9978	
Low-rectal (0∼5cm)	23	35			
Mid-rectal (6∼10cm)	40	26			
Upper-rectal (>10cm)	3	5			
**Type of surgery**					
APR	44	49			
LAR	18	15	0.28		
Others	4	2			
**Chemo-and/or radiotherapy**	23	26	0.61	-0.2204-0.1294	
**Stoma formed**					
No	23	21			
Ileostomy	20	29	0.58		
Colostomy	23	16			
**Resection margin** R0	65	65			
R1	1	1	--		
**Total mesorectal excision**					
Complete	46	38			
Nearly complete	11	17	0.52		
Unknown	3	4			
Incomplete	6	7			

### Postoperative complications and outcomes

The postoperative complications included surgical complications as well as general complications. Surgical complications contain anastomotic leakage [11], ileus, intra-abdominal abscess, urological or perineal wound complications, fistula, hemorrhage and deep vein thrombosis (Table 2). And there were no significant differences between two groups except that laparoscopic group has a significant lower wound complication rate (2 vs 9). General complications include cardiac, respiratory, neurological and renal complications, Ascites etc. Cardiac complications happened in 4 and 3 patients respectively in OpS and LapS groups, containing postoperative heart failure, arrhythmia, angina and ischemic heart diseases, while, the number of patients in the laparoscopic group with respiratory complications was significantly lower (p = 0.03). Notably, the total complication rate between conventional open rectal resection (71 %) and laparoscopic surgery (41%) showed a significant difference (p = 0.0005). 2 cases (3%) in the LapS group were converted to open surgery in the present study.

**Table 2:** Preoperative risk, postoperative complications and other outcomes

Clinical or pathologic feature	OpS (*n*=66)	LapS (*n*=66)	*P*-value
**Preoperative risk**			
**Cr-possum Score**			
∼ 10 percent	29	36	
10 ∼ 20 percent	24	15	0.65
20∼ percent	13	15	
Undergoing re-do surgery	3	2	
Acute or chronic renal impairment	18	12	0.30
Diabetes	13	8	0.34
Cardiac disease	33	21	0.051
Respiratory disease	33	22	0.08
Cerebrovascular disease	2	1	-
**Dindo-Demartines-Clavien Classification**			
Dindo 1	33	41	
Dindo 2	24	9	0.92
Dindo 3	7	14	
Dindo 4	2	2	
**Surgical complications**			
Anastomotic leakage	5	6	-
Prolong ileus	1	1	-
Intra-abdominal abscess	2	1	-
Urological complication	6	5	-
(transurethrale catheter-related problem, urinary tract infection/retension, ureter leakage)			
Perineal wound complication	9	2	**0.03**
(wound dehiscence, wound infections, wound necrosis, abscess or delayed wound healing)			
perforation	1	1	-
Gastrointestinal haemorrhage	2	3	-
Rectal stump abscess	4	1	0.37
DVT	0	1	-
**General complications**			
Cardiac complication	4	3	-
Respiratory complication	10	2	**0.03**
Neurological symptoms	1	0	-
Renal complication	3	0	0.24
Ascites	0	1	-
**Return to normal bowel function**	5.5	4.0	
**30-day mortality**	1	0	-

Cr-POSSUM=Colorectal Physiologic and Operative Severity Score for the enumeration of Mortality and Morbidity. DVT, deep vein thrombosis

### The correlation analysis of Cr-POSSUM and dindo-demartines-clavien classification

There is no significant difference between laparoscopic surgery and conventional surgical procedure in the distribution of Dindo-Demartines-Clavien Classification (p = 0.92). There is a significant positive correlation between open surgery and the Dindo-Demartines-Clavien Classification (Estimate = 0.7495, p= 0.02, 95%CI 1.102∼4.062). In addition, Cr-POSSUM is positively correlated with Dindo-Demartines-Clavien Classification (Estimate = 0.0458, p = 0.01, 95%CI 1.010∼1.085).

### 5-year overall survival, disease-free survival and disease-specific survival rates of different tumor stages and Cr-POSSUM score sub-groups

**Figure 1:**
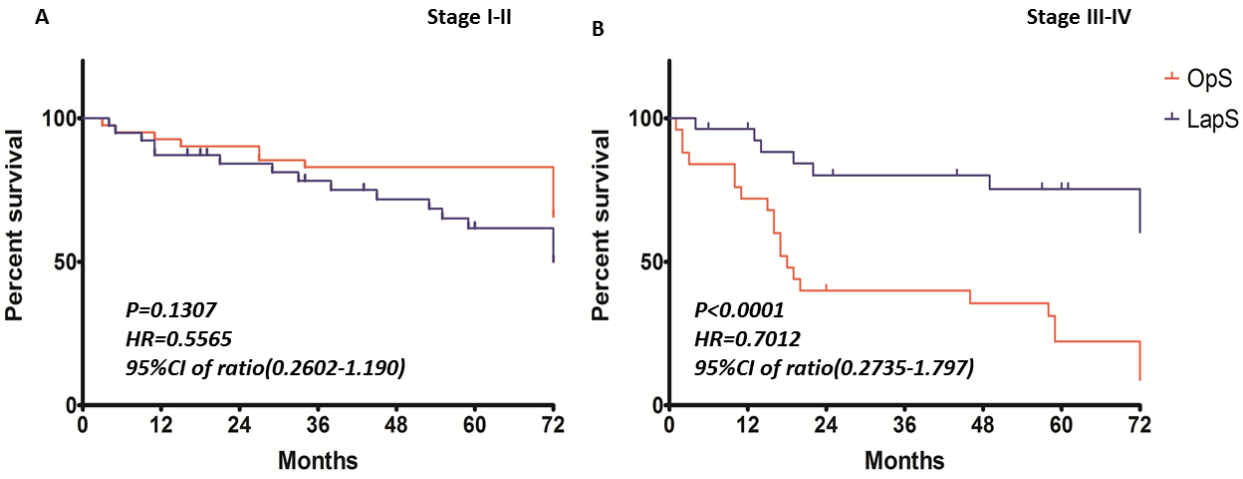
5-year overall survival rates of Different Tumor Stages After Log-rank analysis, no difference could be found between patients undergoing laparoscopic and open rectal resection in stage I∼II (*p* = 0.13, HR 0.5565, 95%CI 0.26-1.19, Figure1A), whereas the overall survival rate was statistically significantly higher in LapS group with stage III-IV tumor (*p* < 0.0001, HR 0.70, 95%CI 0.27-1.79, Figure1B)

The median follow-up is 49.5 months. Using Log-rank analysis, no difference could be found between patients undergoing laparoscopic and open rectal resection in stage I∼II (p = 0.13, HR 0.5565, 95%CI 0.26-1.19), whereas the overall survival rate was statistically significantly higher in LapS group with stage III-IV tumor (p < 0.0001, HR 0.70, 95%CI 0.27-1.79) Figure 1. We further used Cox regression to analyze the 132 patients; it also showed patients undergoing laparoscopic rectal resection had a better overall survival rate.

**Figure 2:**
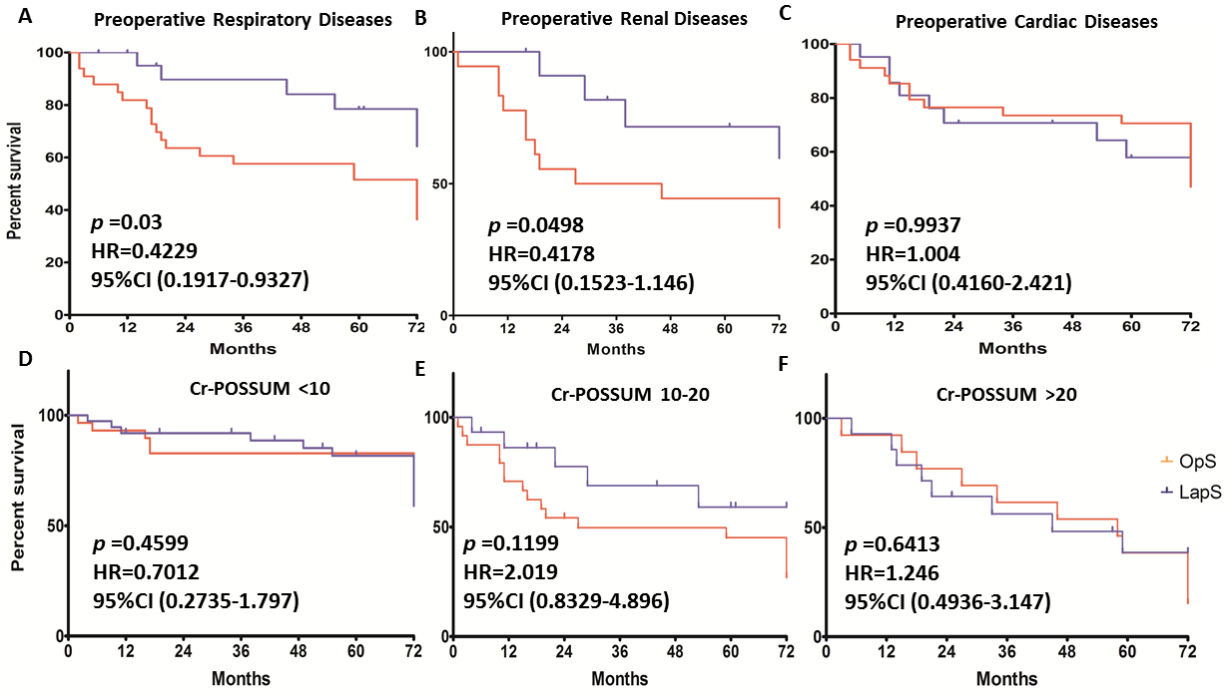
Overall survival rates of patients with preoperative diseases or patients in different Cr-POSSUM score sub-groups **A**.-**C**., the overall survival rates of patients with cardiovascular, pulmonary and renal diseases. **D**.- **F**., The 5- year survival curves of patients in different Cr-POSSUM score sub-groups.

The 5- year overall survival curves of patients in different Cr-POSSUM score sub-groups are shown in Figure 2D, 2E, 2F. The actuarial survivals of the laparoscopic and open groups with Cr-POSSUM valuing 10∼20% was without significantly different (p = 0.12, HR 2.02, 95%CI 0.83-4.90), so was for patients with Cr-POSSUM below 10% (p = 0.46) or above 20% (p = 0.64). The 5-year disease-free survival and disease specific survival are showed in Table 3.

**Table 3:** Disease free survival, disease specific survival and overall survival

Subgroup			5-year survival	HR(95%CI)	*p*
**Stage I∼II**	Disease free survival	LapS	76.7%	0.58(0.28∼1.19)	0.14
		OpS	89.1%		
	Disease specific survival	LapS	74.1%	0.63(0.29∼1.36)	0.24
		OpS	88.6%		
**Stage III∼IV**	Disease free survival	LapS	60%	5.14(2.27∼11.68)	<0.0001
		OpS	38.4%		
	Disease specific survival	LapS	62.1%	5.57(2.42∼12.81)	<0.0001
		OpS	38.4%		
**Preoperative respiratory disease**	Disease free survival	LapS	82.5%	0.40(0.18∼0.87)	0.02
		OpS	64.6%		
	Disease specific survival	LapS	86.4%	0.45(.020∼1.04)	0.047
		OpS	73.3%		
**Preoperative renal disease**	Disease free survival	LapS	80.9%	0.41(0.15∼1.12)	0.049
		OpS	59.9%		
	Disease specific survival	LapS	81.9%	0.38(0.13∼1.15)	0.06
		OpS	61.6%		
**Preoperative cardiac disease**	Disease free survival	LapS	71.9%	0.98(0.41∼2.37)	0.98
		OpS	75.6%		
	Disease specific survival	LapS	75.2%	0.81(0.32∼2.09)	0.68
		OpS	76.9%		
**Cr-POSSUM<10**	Disease free survival	LapS	84.2%	0.68(0.27∼1.70)	0.40
		OpS	89.1%		
	Disease specific survival	LapS	84.2%	2.34(1.08∼5.07)	0.43
		OpS	82.9%		
**Cr-POSSUM 10∼20**	Disease free survival	LapS	73.8%	1.39(0.48∼4.01)	0.54
		OpS	66.5%		
	Disease specific survival	LapS	73.8%	1.28(0.43∼3.78)	0.66
		OpS	68.8%		
**Cr-POSSUM >20**	Disease free survival	LapS	63.6%	1.44(0.54∼3.82)	0.46
		OpS	61.1%		
	Disease specific survival	LapS	72.5%	2.49(0.81∼7.64)	0.11
		OpS	57.6%		
**>75**	Disease free survival	LapS	64.4%	0.89(0.42∼1.89)	0.76
		OpS	60.7%		
	Disease specific survival	LapS	69.4%	0.66(0.29∼1.42)	0.32
		OpS	59.1%		
**<75**	Disease free survival	LapS	62.9%	0.79(0.36∼1.72)	0.55
		OpS	60.8%		
	Disease specific survival	LapS	62.9%	0.79(0.36∼1.72)	0.55
		OpS	60.8%		

### Overall survival, disease-free survival and disease-specific survival rates of patients with preoperative cardiac, renal or respiratory diseases

The overall survival rates of patients with cardiovascular, pulmonary and renal diseases are shown in Figure 2A, 2B, 2C. In patients with the preoperative pulmonary disease, the 5-year overall survival rates of all stages and every different stage in these two groups were significantly different (p = 0.03 [OS], p = 0.02 [DFS]), while in patients with cardiovascular disease, the 5-year overall survivals were not significantly different (p = 0.9). For patients with the preoperative renal disease, the 5-year overall survival rates benefit from laparoscopic surgery with a significant difference. (p = 0.049), however, the disease-specific survival was not significantly different.

**Figure 3:**
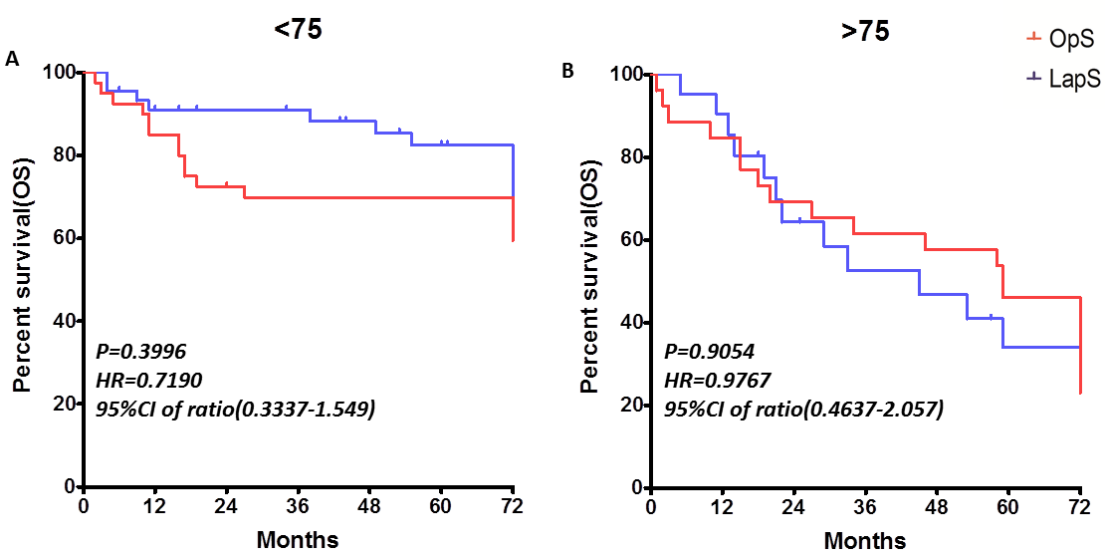
The overall survival rates in age sub-groups (∼75, > 75)

Furthermore, although people older than 75 years account for only 5∼10% of the overall population in developed countries and some developing countries, 35∼45% of patients with rectal cancer are in this age group. This proportion may increase in the future because of demographics of an aging population, and increases in life expectancy [12]. Thus, we separated the patients into two sub-groups (∼75, > 75) by age (LapS 23 of 66 [35 %] > 75y, OpS 26 of 66 [39%] > 75y). The overall survival rate (Figure 3), disease-free survival rate, disease specific survival rate and the complication rate (not show) did not differ significantly in each group.

## DISCUSSION

Recently, the continual innovations of surgical approach are a major step towards the idea of personalized medicine, we should notice that it is still controversial about the treatment strategy for elderly patients with rectal tumor and those with elevated operative risk. Especially for patients with elevated operative risk, patients are most vulnerable when their pre-existing comorbidities make them susceptible to perioperative risk [13, 14, 15].

The COREAN trial demonstrated similar disease-free survival (Lap79.2% vs Open 72.5%) and overall survival rates (Lap 91.7% vs Open 90.4%). The 3-year disease-free survival rate (Lap74.8% vs Open 70.8%) and overall survival rates were similar between both approaches in COLOR II trial as well. More recently, American College of Surgeons Oncology Group [ACOSOG] Z6051 trial [16] and Australasian Laparoscopic Cancer of the Rectum Randomized Clinical Trial [AlaCaRT] [17] investigated the non-inferiority of minimally invasive compared with open pelvic dissection for rectal cancer patients. The results suggest that a laparoscopic resection may not be oncologically justified in many patients requiring protectomy for rectal cancer. However, it was also reported that the follow-up studies to the ACOSOG Z6051 and ALaCaRT trials may show that long-term oncologic outcome are not compromised by a laparoscopic approach and slightly favorable outcomes might be seen as demonstrated by the COREAN and COLOR II trials. Other randomized trials and systematic reviews have also reported that laparoscopic and open proctectomy have similar oncological outcomes [18].However, little solid evidence exists in support of laparoscopic or open proctectomy for patients with high operative risk, although some literature showed that perioperative morbidity did not differ between two groups (Table 4).

**Table 4:** Recent comparative series in advanced rectal cancer

Reference	Year	Lap: Open	Follow up	Stage	Survival	*P* value
**Park *et al***	2009	170:374	36m (2-75)	1-3	3-year DFS lap 77.5%	0.29
					Open 82.6%	
**Laurent *et al***	2009	238:233	52m(1-151)	1-3	5-year DFS lap 82%	NS
					Open 79%	
				3	5-year DFS lap ∼69%	NS
					Open ∼69%	
				3	5-year OS lap ∼72%	0.02
					Open ∼52%	
**Law *et al***	2009	111:310	34m	3	5-year OS lap 56.6%	0.33
					Open 50%	
**Li *et al***	2011	113:123	74.8m	3	5-year OS lap 66.7%	0.85
					Open 70.3%	
				1-3	5-year OS lap 77.9%	0.91
					Open 78.9%	
**Liang *et al***	2011	69:174	Until 3 year	1-3	3-year OS	NS
**Baik *et al***	2011	54:108	Until 5 year	3	5-year OS lap 91.7%	0.30
					Open 77.2%	
				3	5-year DFS lap ∼58.8%	0.63
					Open ∼51.5%	
**Law *et al***	2012	814:1197	40.3m	3	5-year OS lap ∼58%	0.18
					Open ∼48%	
**Park*et al***	2013	404:404	Until 3 years	1-3	5-year OS lap 82.1%	0.44
					Open 81.3%	
				3	5-year OS lap ∼70%	0.26
					Open ∼73%	
				3	5-year DFS lap ∼69%	0.18
					Open ∼59%	
**Asoglu *et al***	2013	513:0	31m(7-64)	3	5-year OS lap ∼70%	-
**Good *et al***^30^	2013	130:0	40m	3	5-year OS lap 75.6%	-
				4	5-year OS lap 53.8%	-
**Ng SS *et al***	2014	136:142	Until 10 years	1-3	10-year OS lap ∼58%	
					Open ∼48%	
				3	10-year RR lap 25.8%	0.08
					Open 43.2%	
**Reibetanz *et al***^29^	2014	170:170	48m vs 46m	1-3	3-year OS	NS
**Bonjer et al** **( COLORII )**	2015	699:345	Until 3 year	1-3	3-year OS lap 86.7%	NS
					Open 83.6%	
					DFS lap 74.8%	NS
					Open 70.8%	
					RR lap 5%	NS
					Open 5%	
**Jeong et al** **(COREAN)**	2015	170:170	Until 3 year	1-4	3-year OS lap 91.7%	NS
					Open 90.4%	
					DFS lap 79.2%	NS
					Open 72.2%	

OS, overall survival; DFS, disease free survival; RR, recurrence rate

It is well accepted that laparoscopic approach is equivalent in the treatment of rectal cancer and shows advantages of shorter hospitalization and faster recovery, lower blood loss and lower complications rates [19], especially in patients with low rectal cancer [20, 21, 22].

Pulmonary comorbidities have been considered as an independent predictor of poor outcome in patients undergoing colectomy and appear to be enhanced in patients with chronic renal diseases. Chronic kidney diseases require dialysis is also a known surgical risk factor that in bowel resection increases the risk of death nearly 6-fold and doubles the complication rate. Therefore, some literature suggests laparoscopic surgery is not attempted for these patients considering their body habitus or longer operative time or creation of pneumoperitoneum which may be potentially associated with adverse pathophysiological changes, including hypercapnia, reduced venous return. However, in this study, patients with preoperative respiratory diseases and renal diseases benefit from laparoscopic surgery, which was consistent with previous reports. The reasons might be lower pain rate, less complication rate in laparoscopic surgery, and also it might be attributable to the enhanced post-operative recovery of lung function in laparoscopic group [23, 24]. Besides, a lung-protective PEEP during pneumoperitoneum might be also valuable for preventing intratidal recruitment/derecruitment [25].

Presently, better preoperative risk assessment should be introduced, objective and accurate evaluation of risk should become routine procedures, those would be helpful to predict and avoid postoperative complications by selecting the appropriate surgical approach. Cr-POSSUM model is a promising specialized tool for monitoring surgical outcomes in colorectal cancer surgery, which might be more accurate than P-POSSUM score [26, 27] in pre-operative use. In present research, patients suffering stage III/IV tumor with a laparoscopic surgery (60%) had primarily a significantly better outcome than patients undergoing open surgery (38.4%), as compared with DFS rates of 64.9% after laparoscopic surgery and 52.0% after open surgery among patients with stage III disease in the COREAN study. There was no significant difference in different Cr-POSSUM subgroups. Other study findings showed that elder patients might benefit most from improved short-term postoperative outcomes following the laparoscopic surgery [28]. Our research did not indicate significant improvements in the overall survival in different age group. The comparable survival rates were reported in series of literature. But the present study showed superior survival in laparoscopic resection, especially in stage III/IV cancers. We reviewed recent researchers: in 2010, the UK MRC CLASICC trial demonstrated that the 5-year overall survival rate (OSR) was 60.3% for laparoscopic rectal resection versus 52.9% for open surgery. Feliciotti´s group [29] (62.5%vs 60.6%), Ng et al [30] (63.9 %vs 55%), Law´s group [31] (71.1%vs 59.3%), Jayne et al [32] (60.3%vs 52.9%) and Baik et al [33] (90.8% vs 88.5%) all presented a better 5-year OSR for laparoscopic rectal resection, though the differences were not significant. Recently, it was reported that laparoscopic resection is associated with more favorable 5-year OS in stage II and III cancer [34, 35]. These results were not influenced by postoperative chemotherapy, which was given similarly after both approaches, especially for stage III cancer. The lower complication rate associated with laparoscopic resection might contribute to the better OS, this reason is more pronounced in the patients with high preoperative risk [36, 37, 38, 39]. Given the increased mortality and morbidity, all efforts should be made to medically optimize these patients preoperatively. One of the limitations of this study is the sample number, though the estimated power was 0.8 (α = 5%). For an instant, only a few patients with diabetes or cerebrovascular diseases were involved in the analyses which still need to be further improved under larger sample amount. Although a randomized controlled trial should be conducted to confirm the findings of the present study, the authors believe that the present study is of value in proposing the future studies.

## References

[1] Kim JG, Heo YJ, Son GM, Lee YS, Lee IK, Suh YJ, Cho HM, Chun CS (2009). Impact of laparoscopic surgery on the long-term outcomes for patients with rectal cancer. ANZ J Surg.

[2] Lujan J, Valero G, Hernandez Q, Sanchez A, Frutos MD, Parrilla P (2009). Randomized clinical trial comparing laparoscopic and open surgery in patients with rectal cancer. Br J Surg.

[3] Agha A, Benseler V, Hornung M, Gerken M, Iesalnieks I, Fürst A, Anthuber M, Jauch KW, Schlitt HJ (2014). Long-term oncologic outcome after laparoscopic surgery for rectal cancer. Surg Endosc.

[4] Quirke P, Steele R, Monson J, Grieve R, Khanna S, Couture J, O’Callaghan C, Myint AS, Bessell E, Thompson LC, Parmar M, Stephens RJ, Sebag-Montefiore D (2009). and MRC CR07/NCIC-CTG CO16 Trial Investigators, and NCRI Colorectal Cancer Study Group. Effect of the plane of surgery achieved on local recurrence in patients with operable rectal cancer: a prospective study using data from the MRC CR07 and NCIC-CTG CO16 randomised clinical trial. Lancet.

[5] Jeong SY, Park JW, Nam BH, Kim S, Kang SB, Lim SB, Choi HS, Kim DW, Chang HJ, Kim DY, Jung KH, Kim TY, Kang GH (2014). Open versus laparoscopic surgery for mid-rectal or low-rectal cancer after neoadjuvant chemoradiotherapy (COREAN trial): survival outcomes of an open-label, non-inferiority, randomised controlled trial. Lancet Oncol.

[6] Bonjer HJ, Deijen CL, Abis GA, Cuesta MA, van der Pas MH, de Lange-de Klerk ES, Lacy AM, Bemelman WA, Andersson J, Angenete E, Rosenberg J, Fuerst A, Haglind E, COLOR II Study Group (2015). A randomized trial of laparoscopic versus open surgery for rectal cancer. N Engl J Med.

[7] Townsley CA, Selby R, Siu LL (2005). Systematic review of barriers to the recruitment of older patients with cancer onto clinical trials. J Clin Oncol.

[8] Feng B, Zhu QL, Xia Y, Lu AG, Wang ML, Li JW, Hu WG, Zang L, Mao ZH, Dong F, Ma JJ, Zheng MH (2010). Direct and indirect costs and long-term survival of laparoscopic anterior resection for rectal cancer. Med Sci Monit.

[9] Zheng MH, Feng B, Hu CY, Lu AG, Wang ML, Li JW, Hu WG, Zang L, Mao ZH, Dong TT, Dong F, Cai W, Ma JJ (2010). Long-term outcome of laparoscopic total mesorectal excision for middle and low rectal cancer. Minim Invasive Ther Allied Technol.

[10] The Royal College of Surgeons of England and Department of Health (2011). Report on the Periop-erative Care of the Higher Risk General Surgical Patient 2011. The Higher Risk General Surgical Patient Towards.

[11] Jannasch O, Klinge T, Otto R, Chiapponi C, Udelnow A, Lippert H, Bruns CJ, Mroczkowski P (2015). Risk factors, short and long term outcome of anastomotic leaks in rectal cancer. Oncotarget.

[12] Manceau G, Karoui M, Werner A, Mortensen NJ, Hannoun L (2012). Comparative outcomes of rectal cancer surgery between elderly and non-elderly patients: a systematic review. Lancet Oncol.

[13] Roviello F, Marrelli D, De Stefano A, Messano A, Pinto E, Carli A (1998). Complications after surgery for gastric cancer in patients aged 80 years and over. Jpn J Clin Oncol.

[14] Sklow B, Read T, Birnbaum E, Fry R, Fleshman J (2003). Age and type of procedure influence the choice of patients for laparoscopic colectomy. Surg Endosc.

[15] Scheidbach H, Schneider C, Hügel O, Yildirim C, Lippert H, Köckerling F (2005). Laparoscopic surgery in the old patient: do indications and outcomes differ?. Langenbecks Arch Surg.

[16] Fleshman J, Branda M, Sargent DJ, Boller AM, George V, Abbas M, Peters WR, Maun D, Chang G, Herline A, Fichera A, Mutch M, Wexner S (2015). Effect of laparoscopic-assisted resection vs open resection of stage II or III rectal cancer on pathologic outcomes: the ACOSOG Z6051 randomized clinical trial. JAMA.

[17] Stevenson AR, Solomon MJ, Lumley JW, Hewett P, Clouston AD, Gebski VJ, Davies L, Wilson K, Hague W, Simes J, ALaCaRT Investigators (2015). Effect of laparoscopic-assisted resection vs open resection on pathological outcomes in rectal cancer: the ALaCaRT randomized clinical trial. JAMA.

[18] Vennix S, Pelzers L, Bouvy N, Beets GL, Pierie JP, Wiggers T, Breukink S (2014). Laparoscopic versus open total mesorectal excision for rectal cancer. Cochrane Database Syst Rev.

[19] Marks JH, Kawun UB, Hamdan W, Marks G (2008). Redefining contraindications to laparoscopic colorectal resection for high-risk patients. Surg Endosc.

[20] Ströhlein MA, Grützner KU, Jauch KW, Heiss MM (2008). Comparison of laparoscopic vs. open access surgery in patients with rectal cancer: a prospective analysis. Dis Colon Rectum.

[21] Mohamed ZK, Law WL (2014). Outcome of tumor-specific mesorectal excision for rectal cancer: the impact of laparoscopic resection. World J Surg.

[22] Salihoglu Z, Baca B, Koksal S, Hakki Hamzaoglu I, Karahasanoglu T, Avci S, Ozben V (2009). Analysis of laparoscopic colorectal surgery in high-risk patients. Surg Laparosc Endosc Percutan Tech.

[23] Stage JG, Schulze S, Møller P, Overgaard H, Andersen M, Rebsdorf-Pedersen VB, Nielsen HJ (1997). Prospective randomized study of laparoscopic versus open colonic resection for adenocarcinoma. Br J Surg.

[24] Asoglu O, Balik E, Kunduz E, Yamaner S, Akyuz A, Gulluoglu M, Kapran Y, Bugra D (2013). Laparoscopic surgery for rectal cancer: outcomes in 513 patients. World J Surg.

[25] Wirth S, Biesemann A, Spaeth J, Schumann S (2017). Pneumoperitoneum deteriorates intratidal respiratory system mechanics: an observational study in lung-healthy patients. Surg Endosc.

[26] Horzic M, Kopljar M, Cupurdija K, Bielen DV, Vergles D, Lackovic Z (2007). Comparison of P-POSSUM and Cr-POSSUM scores in patients undergoing colorectal cancer resection. Arch Surg.

[27] Bromage SJ, Cunliffe WJ (2007). Validation of the CR-POSSUM risk-adjusted scoring system for major colorectal cancer surgery in a single center. Dis Colon Rectum.

[28] Allardyce RA, Bagshaw PF, Frampton CM, Frizelle FA, Hewett PJ, Rieger NA, Smith JS, Solomon MJ, Stevenson AR, Australasian Laparoscopic Colon Cancer Study Group (2010). Australasian Laparoscopic Colon Cancer Study shows that elderly patients may benefit from lower postoperative complication rates following laparoscopic versus open resection. Br J Surg.

[29] Feliciotti F, Guerrieri M, Paganini AM, De Sanctis A, Campagnacci R, Perretta S, D’Ambrosio G, Lezoche E (2003). Long-term results of laparoscopic versus open resections for rectal cancer for 124 unselected patients. Surg Endosc.

[30] Ng SS, Leung KL, Lee JF, Yiu RY, Li JC, Teoh AY, Leung WW (2008). Laparoscopic-assisted versus open abdominoperineal resection for low rectal cancer: a prospective randomized trial. Ann Surg Oncol.

[31] Law WL, Poon JT, Fan JK, Lo SH (2009). Comparison of outcome of open and laparoscopic resection for stage II and stage III rectal cancer. Ann Surg Oncol.

[32] Jayne DG, Thorpe HC, Copeland J, Quirke P, Brown JM, Guillou PJ (2010). Five-year follow-up of the Medical Research Council CLASICC trial of laparoscopically assisted versus open surgery for colorectal cancer. Br J Surg.

[33] Baik SH, Gincherman M, Mutch MG, Birnbaum EH, Fleshman JW (2011). Laparoscopic vs open resection for patients with rectal cancer: comparison of perioperative outcomes and long-term survival. Dis Colon Rectum.

[34] Reibetanz J, Germer CT (2014). Oncological long-term results after open versus laparoscopic surgery for rectal cancer. [Article in German] Chirurg.

[35] Laurent C, Leblanc F, Wütrich P, Scheffler M, Rullier E (2009). Laparoscopic versus open surgery for rectal cancer: long-term oncologic results. Ann Surg.

[36] Park IJ, Choi GS, Lim KH, Kang BM, Jun SH (2009). Laparoscopic resection of extraperitoneal rectal cancer: a comparative analysis with open resection. Surg Endosc.

[37] Reibetanz J, Germer CT (2014). [Oncological long-term results after open versus laparoscopic surgery for rectal cancer]. Chirurg.

[38] Good DW, O’Riordan JM, Moran D, Keane FB, Eguare E, O’Riordain DS, Neary PC (2011). Laparoscopic surgery for rectal cancer: a single-centre experience of 120 cases. Int J Colorectal Dis.

[39] Schwenk W, Böhm B, Witt C, Junghans T, Gründel K, Müller JM (1999). Pulmonary function following laparoscopic or conventional colorectal resection: a randomized controlled evaluation. Arch Surg.

